# Numerical Analysis of Fracture Behaviour for Cracked Joints in Corrugated Plate Girders Repaired by Stop-Holes

**DOI:** 10.3390/ma16103606

**Published:** 2023-05-09

**Authors:** Qifei Wang, Wenfeng Zhou, Zhiyu Wang, Sijie Xiang, Guowen Yao, Qunyi Huang, Yinggai Liu

**Affiliations:** 1 Key Laboratory of Deep Underground Science and Engineering (Ministry of Education), School of Architecture and Environment, Sichuan University, Chengdu 610065, China; 2 CREGC Architectural & Construction Engineering Co., Ltd., Chengdu 610031, China; 3 State Key Laboratory of Mountain Bridge & Tunnel Engineering, Chongqing Jiaotong University, Chongqing 400074, China; 4 College of Civil Engineering, Southwest Jiaotong University, Chengdu 610031, China

**Keywords:** fracture, cracked joint, stress intensity factor, corrugated plate girder, stop-hole, numerical analysis

## Abstract

The efficient crack eliminated stop-hole measure was proposed to repair and reduce the stress concentration associated fracture risk of the corrugated plate girders by setting it at the critical joint of flange plate with tightened bolts and gaskets under preloading. To investigate the fracture behaviour of these repaired girders, parametric finite element analysis was conducted, focusing on the mechanical feature and stress intensity factor of crack stop-hole in this paper. The numerical model was verified against experimental results first, and then the stress characteristics due to the presence of crack open-hole were analysed. It was found that the moderate-sized open-hole was more effective than the over-sized open-hole in the reduction of stress concentration. For the model with prestressed crack stop-hole through bolt preloading, the stress concentration was nearly 50% with the prestress around open-hole increased to 46 MPa, but such a reduction is inconspicuous for even higher prestress. Relatively high circumferential stress gradients and the crack open angle of oversized crack stop-holes were decreased owing to additional prestress effects from the gasket. Finally, the shift from the original tensile area around the edge of the crack open-hole that was prone to fatigue cracking to a compression-oriented area is beneficial for the reduction of stress intensity factor of the prestressed crack stop-holes. It was also demonstrated that the enlargement of crack open-hole has limited influence on the reduction of stress intensity factor and crack propagation. In contrast, higher bolt prestress was more beneficial in consistently reducing the stress intensity factor of the model with the crack open-hole, even containing long crack.

## 1. Introduction

Corrugated plate girders have been used in bridge engineering for several decades, and their application may have various cross-sectional characters from conventional I or H section [1,2] to composite box section composed of concrete and steel in mostly steel girder-concrete slab composite bridges [3,4]. During the service life of the bridge, these girders often experience varied degrees of deterioration, especially due to fatigue loading [5]. The stress existing in the joints of corrugated plate girders is complex, leading to severe stress concentration problems [6], especially for the web-flange weld toe stress affected by web corrugation geometry and flange. As a result, the fracture of the web-flange welds has been commonly observed for these girders, as reported in Refs. [7,8]. Although the fatigue design curve of the Category B’ of the AASHTO LRFD Bridge Design Specifications [9] was demonstrated to be suitable in the design of the fatigue life of this kind of girder [10], there has been still been no consensus among recent research work (e.g., Refs. [11,12,13]) on how to further eliminate fatigue cracks and improve the fracture resistance of the web-flange welds in these girders.

As a temporary and emergency treatment in the maintenance of steel structures, the crack stop-hole measure has a prominent advantage to eliminate the fatigue cracks induced by stress concentration at the crack tip and thus to extend the structural fatigue life [14]. When the crack propagation occurs, the inherent crack arresting behaviour of the stop-holes is influenced by the hole geometries and the drilling defects. Most early research work has been performed to examine the influence of the hole diameter under tension and combined bending and shear. For example, Chen [15] determined an appropriate size of a stop-hole for temporarily repairing cracked marine and offshore structures in which a rational choice of stop-hole size is affected by not only the size and nature of the crack but also by the additional conservatism. Jiang et al. [16] compared the performances of unrepaired and repaired specimens with a stop-hole to conclude that the larger the hole diameter, the smaller the stress concentration factor and fatigue notch coefficient by drilling holes. Additionally, the hole diameter of 14 mm was proposed for the case in their study when considering the overweakening of the section rigidity. Song and Shieh [17] showed that the larger the stop-hole diameter, the longer the crack initiation and total fatigue lives for specimens of 6061-T651 aluminium alloy and AISI 304 stainless steel. Yao et al. [18] studied the maintenance effect of stop-hole approach for cracks at diaphragm-to-rib weld. It was found that the stress characteristics around the stop-hole were out of the ordinary and larger stop-hole diameter could significantly alleviate stress concentration and delay the crack propagation for the welds under out-of-plane deformation. Ayatollahi et al. [19,20] studied the drilling a hole at the crack tip, turning the crack into a notch and diminishing the crack tip stress singularity. Their study showed that a higher hole diameter resulted in a higher life extension for crack repair using stop-hole technique under pure mode-I and pure mode-II loading conditions. The larger hole diameters also resulted in lower stress values at the hole edge causing the fatigue crack to initiate over a longer time. 

The crack arrest behaviour of the stop-holes can be improved through optimizing the hole shape [21] and the arrangement, and by adjusting from the flank hole in front of the crack tip [22] to symmetric crack flank holes along the crack flanks [23] with different hole position. For example, Lu et al. [24] studied the fatigue crack growth behaviour with the pure stop-hole effect and the combination of stop-hole and overload. It was found that the stop-hole diameter had a positive influence on the fatigue life, and the stop-hole location had a negative influence on fatigue life since the relatively extended fatigue life decreases linearly as the ratio of the stop-hole location to the width of the specimen increases. Razavi et al. [25] experimentally and numerically studied the effects of double stop-hole method on the fatigue life extension of SENT specimens made of S690 steel alloy. The stress condition around the stop-hole was shown to be considerably influenced by the arrangement and size of the stop-holes since lower stress concentration around the double stop-hole resulted in higher fatigue crack initiation life. Makabe et al. [26] conducted experiments to demonstrate that, in the right place, a stop-hole could alter the growth direction of a crack when multiple cracks were present in the material [27], thereby preventing their joining together. The benefits from crack stop-hole in changing the crack growth direction are also influenced by additional hole locations [28]. The crack-stopping properties of any additional holes is dependent on their diameters and locations [29]. The use of prestress of the high-strength bolt in reducing the tensile stress around the crack stop-hole was also reported in Refs. [16,30]. 

Despite significant research mentioned above, there is little research in the literature regarding the crack arrest behaviour of the joints in corrugated plate girders. Previous studies [11,13] have shown that the fracture is likely to take place for cracked joints in corrugated plate girders, but the development law remains unclear when the repair of these joints using advantageous crack stop-hole measure is concerned. Therefore, it is necessary to develop an in-depth numerical modelling to further the understanding of the influence of related geometric parameters on the fracture resistance of the joints in the girders. 

Moreover, in view of expensive and time-consuming experimental parametric studies, the reasonable accuracy of the FE model in reproducing the experimental behaviour of cracked joint details makes it a useful model for both analytical and parametric studies, especially when the stress intensity factor is concerned. Therefore, this work studies the fracture behaviour of corrugated plate girders repaired by crack stop-holes. The numerical model is firstly validated against presented experimental stress data, and the effects of the crack stop-hole parameters on the stress concentration and the stress intensity factor are analysed to establish the relationship of the fracture between the unrepaired cases and the repaired cases. Finally, the mechanism of the prestressed crack stop-holes for the reduction of stress intensity factor was discussed. 

## 2. Research Method and Model Development

### 2.1. Description of Experimental Model

**Figure 1:**
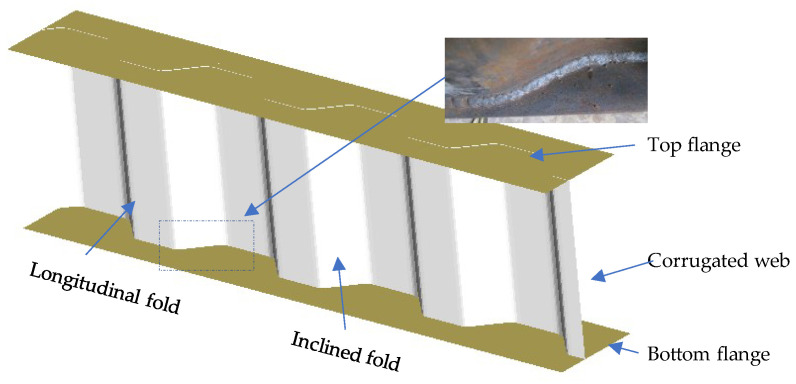
Schematic diagram of corrugated plate girder.

**Figure 2:**
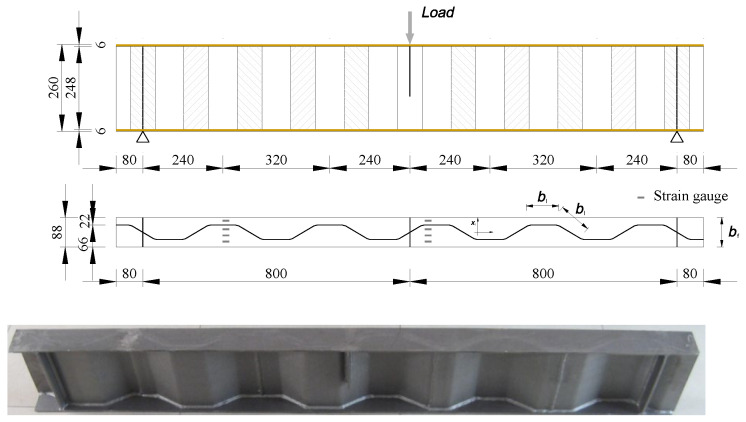
Dimensions of the test corrugated plate girder (unit: mm).

The authors [11] carried out an experimental study on the fatigue behaviour of corrugated plate girders under three-point loading and the girder configuration with the corrugated web welded to the flange is schematically shown in Figure 1. The specimen was 1760 mm in length and its top and bottom flange plates were 6 mm thick and 88 mm wide. The longitudinal fold and the inclined fold of the corrugated web are of the same widths of 82 mm, as shown in Figure 2. The mechanical properties of steel plate and welding rod in the test specimen are listed in Table 1 with the standard derivation of 10.87 MPa corresponding to 5% probability. The fatigue load was applied following BS7608 [31] at the centre of span in form of sinusoidal wave with constant frequency of 3 Hz to eliminate the unfavourable influence of the frequency on fatigue within the range of statistical variation as suggested in Ref. [32]. The applied stress ratio was set at 0.1 and the minimum load was 8 kN in consecutive loading until failure. The load range was selected to ensure that the local instability of the top flange in compression was prevented and all the structural components of the girder were in an elastic state. The facilities and process details of fatigue testing can be referred to in Ref. [11]. 

### 2.2. Finite Element Model Construction

As concerns the loading-bearing capacity, both test specimens broke with crack propagation of the bolt shank from the screw thread root after flexural bending. It is necessary to understand the flexural bending capacity of the fracture section close to the contact surface between the bolted sphere and the sleeve. A theoretical basis was also presented for the fracture-related stress intensity factor and for the test-bolted spherical joints in the following.

**Figure 3:**
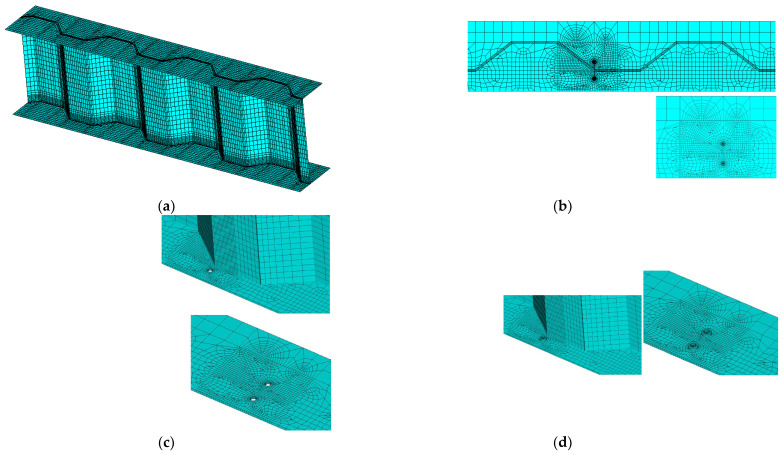
Illustration of meshes in FE model. (**a**) Model overview; (**b**) Introduction of surface crack into joint; (**c**) Repair case i with crack stop-holes; (**d**) Repair case ii with pre-stressed crack stop-holes.

**Figure 4:**
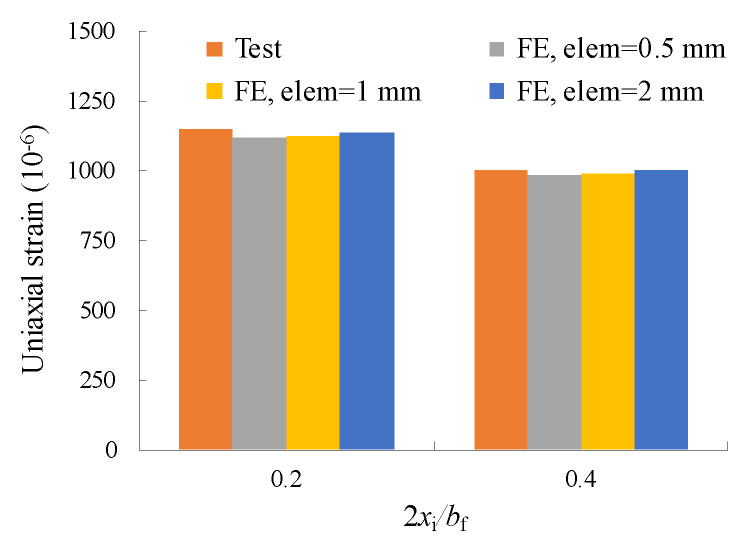
Comparison of test and modelling strain data.

An identical three-dimensional test corrugated plate girder was simulated with the aid of ANSYS software version 17.0. Only half of the model was developed due to symmetry in configuration, and its overview is shown in Figure 3a. The steel material was assumed to be linear elastic until fracture. The top and bottom flanges were modelled by a 20-node solid element (Solid 186) which was able to exhibit quadratic displacement behaviour. Meanwhile, the corrugated web was modelled by a four-node element (Shell 181) with six degrees of freedom at each node. The material modulus of elasticity and Poisson ratio were taken as *E* = 206 GPa and *ν* = 0.3, respectively, following Ref. [9] in a perfect elasto-plastic material model. The modelled girder was simply supported at the end, leaving the rotation about the line unsupported. To replicate the fracture behaviour, a surface crack was introduced at the joint between the longitudinal fold and the inclined fold of the corrugated web, as plotted in Figure 3b. The crack body containing the singularities in front of two crack tips and a seam in between. All related stress concentration was properly simulated using an isoperimetric crack tip element in an initial input file aided processor. Furthermore, two repair cases with crack stop-holes were considered for the purpose of contrast, i.e., Repair case i concerning the crack stop-hole covering the crack tip as shown in Figure 3c and Repair case ii concerning the bolt prestressed crack stop-hole adjoining bolt gasket under the bolt preloading, as shown in Figure 3d. The following analysis was performed for the models with the existence of the crack seam. Based on a convergence and mesh independence study, the general element size was set as 2 mm and, with a very fine mesh of 0.5 mm, was considered around the crack front. Less variability of the chosen mesh size on the local uniaxial stress gradient can be observed from a mesh sensitivity analysis when the modelling strain results are compared against experimental measurement related to the monotonic test as shown in Figure 4. It was determined to refine the mesh to 0.5 mm near the crack region and the crack stop-hole while relatively dense mesh was applied to other lower stress regions. Additionally, the finite element model was not developed for lifetime simulation but for constant stress and strain conditions. The computational time required to perform the finite element analysis of the corrugated plate girder model with the joint crack at one loading case was approximately 0.5 h using a computer with the following parameters: Core i7 Processor + 2.20 GHz with DDR 3–8 GB RAM. 

### 2.3. J Integral-Based SIF Analysis

Since the corrugated plate girders subjected to bending are usually designed considering that only the flanges resist the tension force [2], the bottom flange plate is mostly subjected to the crack opening mode, i.e., mode I. This mode is featured by the displacement at the lips of the crack are perpendicular to the crack propagation. For the two-dimensional medium loaded in mode I, *K*_I_ in mode I can be determined by the development for finite pieces with cracks in practical application [32] as:

(1) $$K_{I}=\sigma \sqrt{\pi a}\cdot Y(a)$$

where *Y*(*a*) is a dimensionless number geometric correction factor depending on the geometry of piece and the crack length. When the crack is very small in relation to the piece’s dimension, *Y*(*a*) approaches 1.0. Otherwise, it can be correlated as a function of geometry with the crack length a and the stress tensor at a distance with a half crack length. Finite element (FE) modelling can be used to describe the crack front in the linear elastic domain. With respect to the elastic strain energy density, *W*, and the traction vector, *T*, the original formulation for integral *J* in the elastic cracked plane in the absence of crack loading can be expressed following Ref. [32] as:

(2) $$J=\int_{C}Wdy-\bar{T}\cdot \partial \bar{u}/\partial x\cdot dS$$

where *u* is the displacement vector. *C* is the contour that runs counter clockwise from the lower crack surface to the upper crack surface. Any continuous *C* from the interior crack’s fracture tip to the superior crack’s edge is the subject of the integral. *T*_i_ is the tension vector on *C*, and its direction toward *C* is determined by the normal vector [32]. Based on Equation (2) and the arrangement in ANSYS general post-processor, showing how much energy is delivered per unit area of break surface increment, *J*, this can be obtained by taking the math mean of the strain energy results following a few different concentric way lengths around the break tip. For the joint of the corrugated plate girder examined here, the corresponding SIF in the linear elastic state [32] can be obtained as *K*_I_ = √(*J*·*E*).

## 3. Results and Discussion

### 3.1. Experimental Results

**Figure 5:**
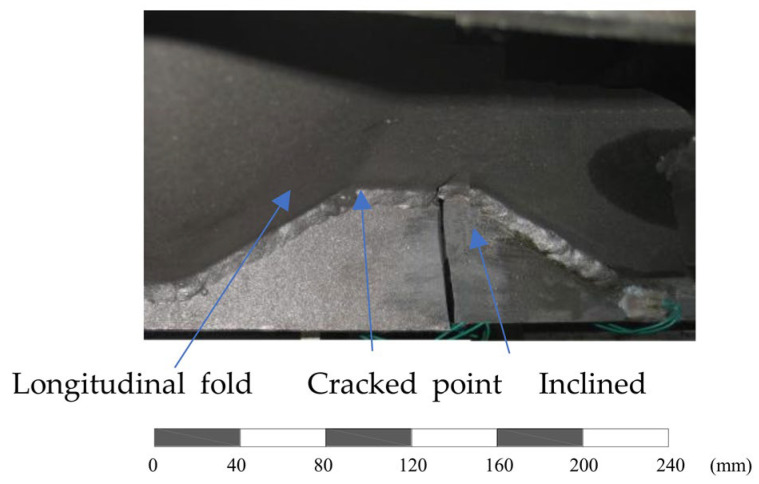
Fracture mode of the test joint.

The test specimen was seen to suffer ultimate fracture after 1,048,300 loading cycles at the joint between the longitudinal fold and the inclined fold of the corrugated web. The direction of the crack propagation is almost perpendicular to the longitudinal stress direction after the fracture, as shown in Figure 5 with a scale bar in mm. The measured uniaxial strains at the bottom flange for the cracked joint (2 *x*_i_/*b*_i_ = 0.4) are obviously greater than that near the centreline of the flange (2 *x*_i_/*b*_i_ = 0.2), as shown in Figure 4. 

### 3.2. Numerical Verification

The comparison of test and modelling strain in Figure 4 shows good consistency. Although the test measured strains slightly declined with the increase of the number of cycles, the difference between test strain data and modelling constant strains as the mean value was kept within 5%. Moreover, the same accuracy can be observed for the measured points when 2 *x*_i_/*b*_i_ is varied from 0.2 to 0.4.

**Figure 6:**
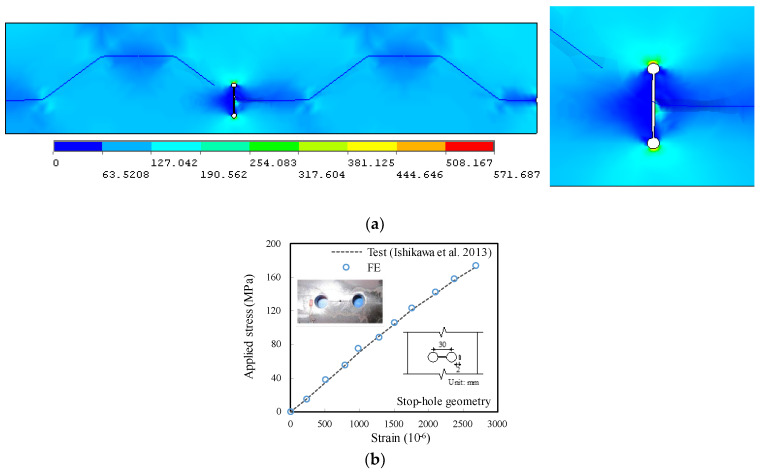
Comparison of typical stress feature of un-prestressed crack-stop hole. (**a**) Principal stress distribution; (**b**) Applied stress–strain verification for referred test [33].

The numerical principal stress contours of the finite element model for the unrepaired case without crack stop-hole are shown in Figure 6a. An obvious peak stress of 571.68 MPa close to the load carrying capacity took place at the prolongation seam to the edge of the crack stop-hole, making the position become the potential stress concentration point at the joint between the longitudinal fold and the inclined fold of the corrugated web. In terms of trend, it appears that fatigue cracks are likely to initiate and generate at such point of the hole edge and propagate further, which is in good agreement with the observation of fractured joint, as shown in Figure 4. The experimental applied stress–strain relation for similar crack stop-hole details reported in Ref. [33] was also referred for verification. The strain in this referred test was measured through a strain gauge mounted at 2 mm from the edge of the stop-hole, which was also demonstrated to be the maximum in the modelling, as plotted on the right side of Figure 6a. As shown in Figure 6b, the modelling and referred experimental applied stress–strain relations were in good correlation, which indicates that the developed finite element model was also able to capture the linear increase of the critical stress at the stop-hole location. 

**Figure 7:**
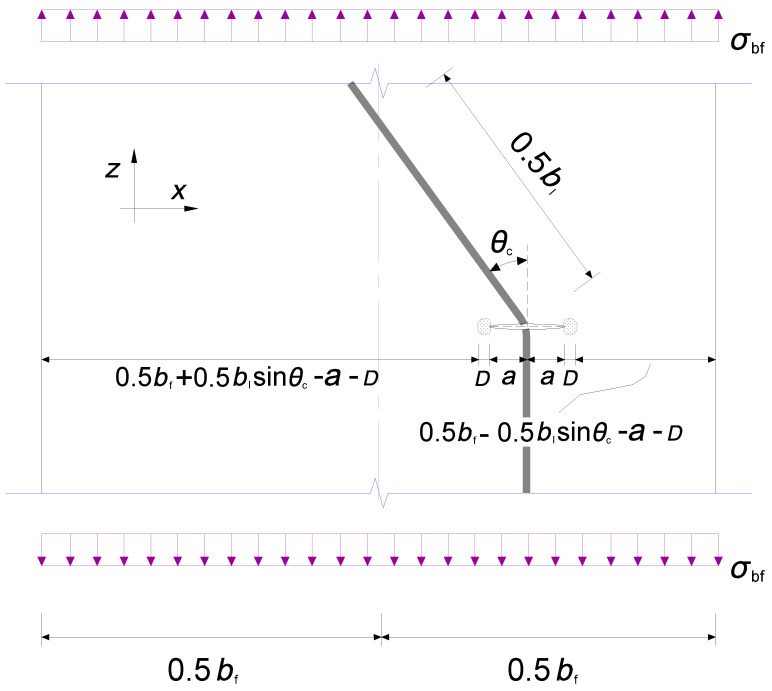
Joint geometric relation around the crack tip.

For the sake of interpreting the stress state around the crack front of the cracked joint, suppose the centre of the crack coincided with the centreline of the longitudinal fold of the corrugated web adjoining the flange plate, as shown in Figure 7. The crack tip is represented by a half-length (*a*) under remote tensile stress on the flange *σ*_bf_ referring to a similar finite width plate model in Ref. [34]. The equilibrium of tensile stress along the *z* axis for two crack tips and a seam in between can be written as:

(3) $$\int_{0}^{D_{l}}\frac{\gamma_{1}\sigma_{{}_{\mathrm{bf}}}a^{0.5}}{{\left(2r\right)}^{0.5}}dr+\gamma_{1}^{2}\sigma_{{}_{\mathrm{bf}}}(0.5b_{f}+e-D_{l}-a)+\int_{0}^{D_{r}}\frac{\gamma_{2}\sigma_{{}_{\mathrm{bf}}}a^{0.5}}{{\left(2r\right)}^{0.5}}dr+\gamma_{2}^{2}\sigma_{{}_{\mathrm{bf}}}(0.5b_{f}+e-D_{r}-a)=\sigma_{{}_{\mathrm{bf}}}b_{f}$$

where *e* is the eccentricity between the centre of the crack and the centreline of the flange while it can be deduced as 0.5 *b*_l_sin*θ*_c_. *D* is the controlled radius of SIF dominance zone around the crack tip in which the left and the right counterparts are denoted as *D*_1_ and *D*_2_, respectively. *Γ*_1_ and *γ*_2_ are the correction variables. 

Meanwhile, the equilibrium of bending moment with respect to the centreline of the flange can be given by:

(4) $$\int_{0}^{D_{l}}\frac{\gamma_{1}\sigma_{{}_{\mathrm{bf}}}a^{0.5}}{{\left(2r\right)}^{0.5}}dr(D_{l}+a-e)+0.5\gamma_{1}^{2}\sigma_{{}_{\mathrm{bf}}}\left[0.25b_{\mathrm{bf}}^{2}-{\left(D_{l}+a-e\right)}^{2}\right]=\int_{0}^{D_{r}}\frac{\gamma_{2}\sigma_{{}_{\mathrm{bf}}}a^{0.5}}{{\left(2r\right)}^{0.5}}dr(e+D_{r}+a)+0.5\gamma_{2}^{2}\sigma_{{}_{\mathrm{bf}}}\left[0.25b_{\mathrm{bf}}^{2}-{\left(D_{r}+a+e\right)}^{2}\right]$$

Solving Equations (3) and (4) yields:

(5) $$\gamma_{1}^{2}\left[0.25b_{\mathrm{bf}}^{2}-{(a-e)}^{2}\right]-\gamma_{2}^{2}\left[0.25b_{\mathrm{bf}}^{2}-{(a+e)}^{2}\right]=3ae$$

Therefore, the aforementioned geometric correction factor in Equation (1) for this simple model can be theoretically expressed as the maximum value of *γ*_1_ and *γ*_2_ as:

(6) $$Y(a)={\left(\frac{b_{f}-2e-a}{b_{f}-2e-2a}\right)}^{0.5}={\left[\frac{1-b_{l}\sin\theta_{c}/b_{f}-a/b_{f}}{1-b_{l}\sin\theta_{c}/b_{f}-2a/b_{f}}\right]}^{0.5}$$

**Figure 8:**
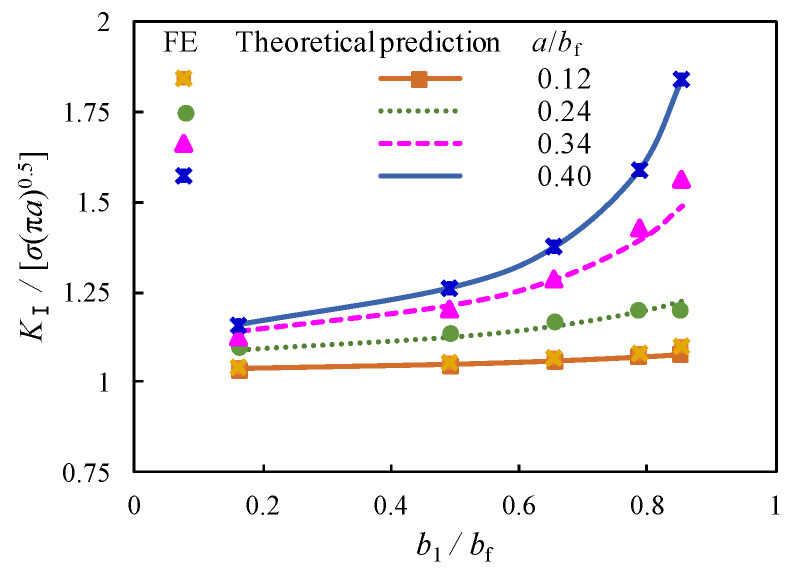
Comparison of normalized *K*_I_ predicted by theoretical calculation and modelling.

For the purpose of verification, the *K*_I_ from finite element modelling is normalized by *σ*_bf_√(π·*a*), which is reasonably consistent with theoretical calculations for the relation of b_1_/b_f_ from Equation (6) in Figure 8. The relative error defined by *K*_I_ from finite element modelling (*K*_I,FE_) divided by that from theoretical prediction (*K*_I,Theoretical_) is listed in Table 2. The maximum error can be seen for *K*_I,FE_/*K*_I,Theoretical_ when *b*_l_/*b*_bf_ = 0.85 is the maximum eccentricity between the centre of the crack and the centreline of the flange. In this case, the crack is closer to the edge of the flange plate which produces greater destruction of the plate. Notwithstanding this, all the list errors are within 5% difference. 

### 3.3. Parametric Study

For corrugated plate girders repaired by stop-holes, how to quantify the stress characteristics is the concern of this work. The stress field around stop-holes are described by the known stress concentration factors and stress intensity factors which are expected to be influenced by the geometric parameters listed in Table 3 related to the longitudinal fold and the crack stop-hole and the load parameters of the bolt prestress as referred from FHWA [35]. Therefore, based on the validated finite element model, these influences on the fracture behaviour of corrugated plate girders repaired by stop-holes were analysed as below.

#### 3.3.1. Comparison of Stress Concentration

**Figure 9:**
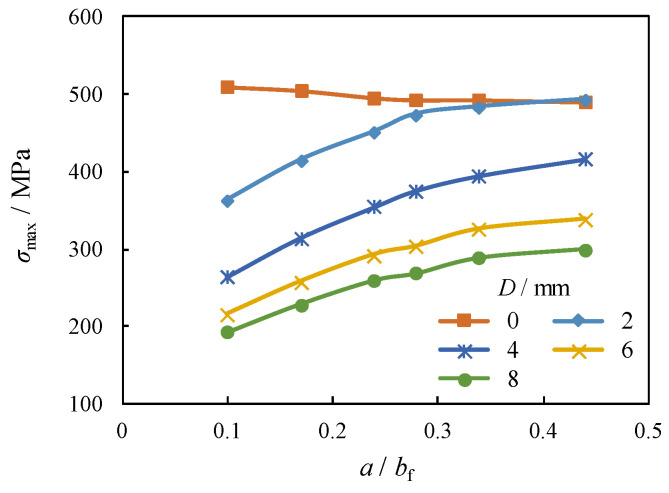
Comparison of *σ*_max_ with varied *a*/*b*_f_.

With the aforementioned verified numerical model, models of repaired corrugated plate girders with the diameters of crack stop-holes of 2 mm, 4 mm, 6 mm, and 8 mm were built and the distribution of the maximum principal stress (*σ*_max_) around crack stop-hole for the repaired case was compared with an unrepaired case without crack stop-hole in Figure 9. The longitudinal fold width is set as the same as the test, i.e., 82 mm. 

**Figure 10:**
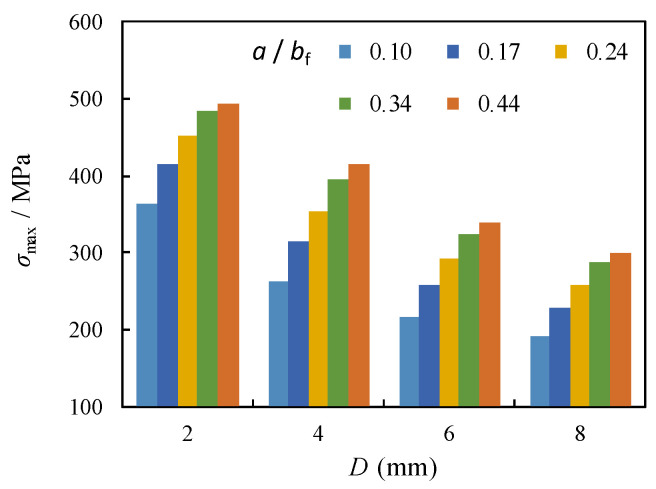
Comparison of *σ*_max_ with varied *D*.

It can be indicated from the plotted comparison that the *σ*_max_ for the unrepaired case (i.e., *D* = 0) remains almost unchanged when the crack propagation in the flange (i.e., *a*/*b*_f_) shifts from 0.1 to 0.44. In contrast, *σ*_max_ around the crack stop-hole for the repaired case i is significantly amplified with the increase of *a*/*b*_f_, as shown in Figure 10. Moreover, the increment of *σ*_max_ becomes decelerated with larger *a*/*b*_f_. By adopting the same comparison, the *σ*_max_ around crack stop-hole is notably reduced with the increase of diameter of crack stop-hole (*D*) from 2 mm to 4 mm, e.g., 26% and 18% decrement for the *a*/*b*_f_ = 0.3 and 0.4, respectively. Such a decrease becomes less obvious for a further increase of *D* from 6 mm to 8 mm, e.g., 13% and 12% decrement for the *a*/*b*_f_ = 0.3 and 0.4, respectively. The crack arrest effect of the crack stop-hole for the repaired case was further evaluated by the stress concentration factor (*K*_t_), which is defined by:

(7) $$K_{t}=\sigma_{\max}/\sigma_{\mathrm{nom}}$$

(8) $$\sigma_{\mathrm{nom}}=F/\left[(b_{f}-2D-2a)t_{f}\right]$$

where *σ*_nom_ is the nominal stress in the flange plate with a crack stop-hole.

**Figure 11:**
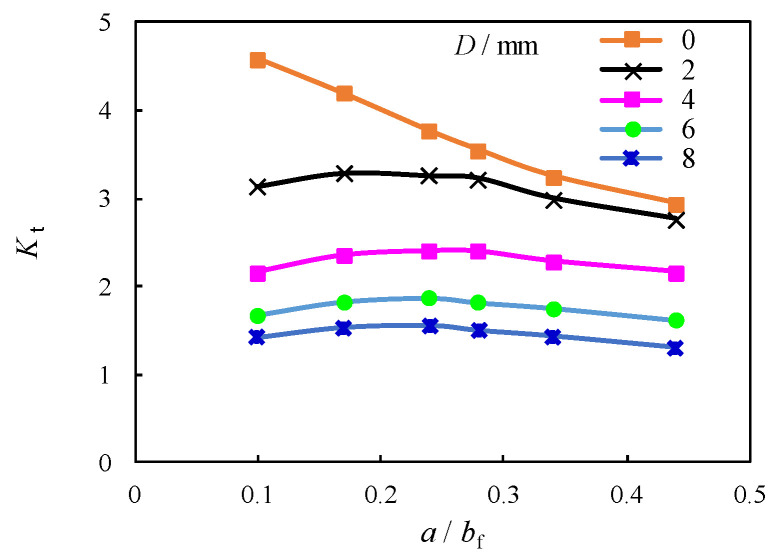
Relation between *K*_t_ and *a*/*b*_f_.

**Figure 12:**
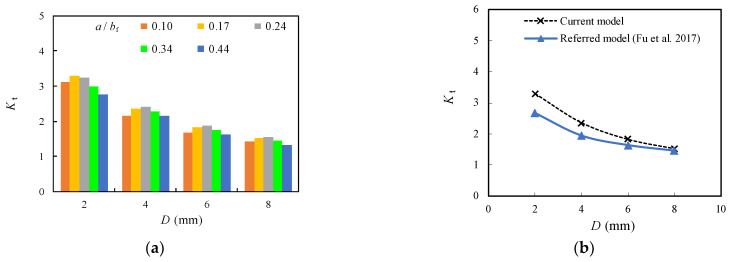
Relation between *K*_t_ and *D.* (**a**) Contrast of *a*/*b*_f_; (**b**) Contrast with referred experimental model [36].

From the perspective of the trends shown in Figure 11, it was evidenced that *K*_t_ was obviously reduced for the unrepaired case (i.e., *D* = 0) while becomes less sensitive to change for all models with crack stop-hole for the repaired case i. This can be due to the elimination of the plastic regions near the crack tip with the introduction of crack stop-hole. A closer examination in Figure 12a indicates that *K*_t_ can be further decelerated with the increase of the diameter of crack stop-hole (*D*). The increase of the diameter of crack stop-hole (*D*) from 2 mm to 4 mm results in 34% and 27% decrement of *K*_t_ for the model with *a*/*b*_f_ = 0.3 and 0.4, respectively. Such a decrease becomes less obvious for the further increase of *D* from 6 mm to 8 mm, e.g., 21% and 23% decrement for the *a*/*b*_f_ = 0.3 and 0.4, respectively. Theoretically, if an oversized crack stop-hole is adopted for the repaired case, the stiffness of the section is greatly reduced, and excessive weakening induced failure will occur. According to the findings in [36] for compact tension experimental model for the deck with a similar configuration in this study, *K*_t_ at the crack tip decreases with the increase of *D*. The resultant downward change trend with larger *D* is shown to be gradually consistent with the findings for the current model presented in this study, as shown in Figure 12b, although the *K*_t_ predicted by the current model is 10% higher than the referred model. 

**Figure 13:**
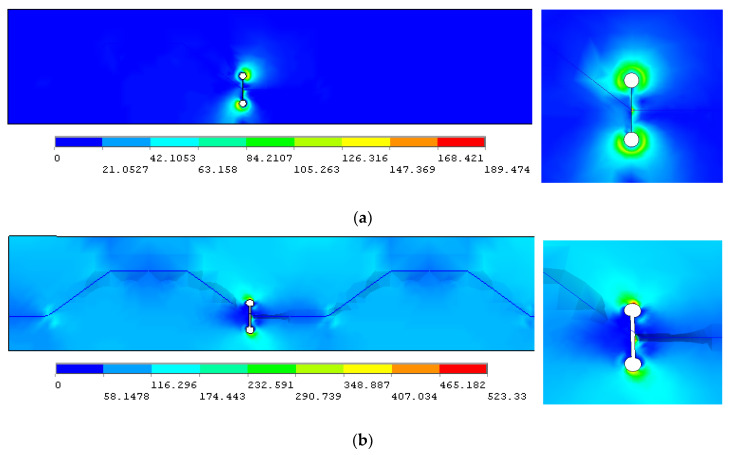
Principal stress distribution near prestressed crack stop-hole. (**a**) Pre-stressing around crack stop-hole; (**b**) Crack face separation under *σ*_bf_.

For the repair case ii, the area around crack stop-hole was firstly stressed under the prestress of the gasket from the high-strength bolt (*σ*_bt_ = 46 MPa), as shown in Figure 13a, and was then subjected to the longitudinal tensile stress *σ*_bf_ on the bottom flange, as shown in Figure 13b. The principal stress contour is more evenly distributed near the joint between the longitudinal fold and the inclined fold of the corrugated web of the girder when comparing to Figure 6. Moreover, the maximum principal stress around crack stop-hole was shown to be obviously reduced in Figure 13b and transferred from the outer crack stop-hole near the plate edge for the repaired case ii to the inner crack tip for the repaired case i. This can be expected as the compression induced by *σ*_bt_ partly offset the tension induced by *σ*_bf_, which in turn slows the fatigue crack growth of the outer crack stop-hole on the bottom flange.

**Figure 14:**
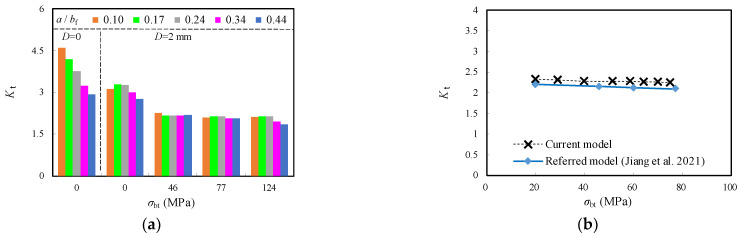
Relation between *K*_t_ and *σ*_bt_. (**a**) Contrast of *a*/*b*_f_; (**b**) Contrast with referred experimental model [16].

To better understand the effect of *σ*_bt_ on the stress concentration, the models with *D* = 2 mm were developed since their corresponding *K*_t_ was greater than others with increased diameters of crack stop-holes, as plotted in Figure 12a. As shown in Figure 14a and Table 4, *K*_t_ was obviously reduced by nearly 50% with the increase of *σ*_bt_ from 0 to 46 MPa, which can be approximated to be 45~60% of *K*_t_ for the original model with *D* = 0 mm. However, the further increase of *σ*_bt_ from 46 MPa to 144 MPa has inconspicuous effect on the reduction of *K*_t_ related stress concentration around crack stop-hole, which requires a specific analysis of the relation between the stress gradient and local geometric parameters. A further contrast with the findings of referred experimental model [16] is shown in Figure 14b. A very close trend with error within 5% can be seen for the downward change trend, with increased *σ*_bt_ from 20 MPa to 77 MPa. 

#### 3.3.2. Comparison of Critical Circumferential Stress Gradient Localized around the Stop-Hole

**Figure 15:**
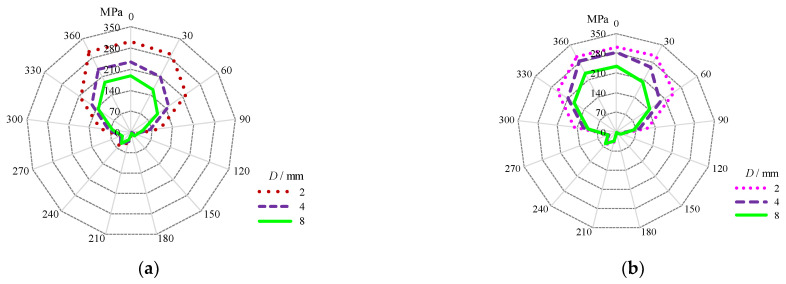

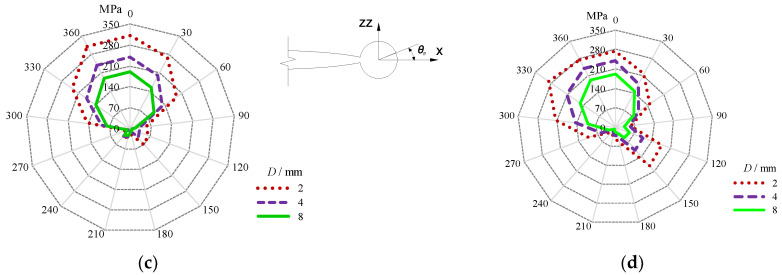
Principal stress distribution at the perimeter of crack stop-hole. (**a**) *a*/*b*_f_ = 0.12 without bolt prestress; (**b**) *a*/*b*_f_ = 0.2 without bolt prestress; (**c**) *a*/*b*_f_ = 0.12 with *σ*_bt_ = 46 MPa; (**d**) *a*/*b*_f_ = 0.12 with *σ*_bt_ = 124 MPa.

**Figure 16:**
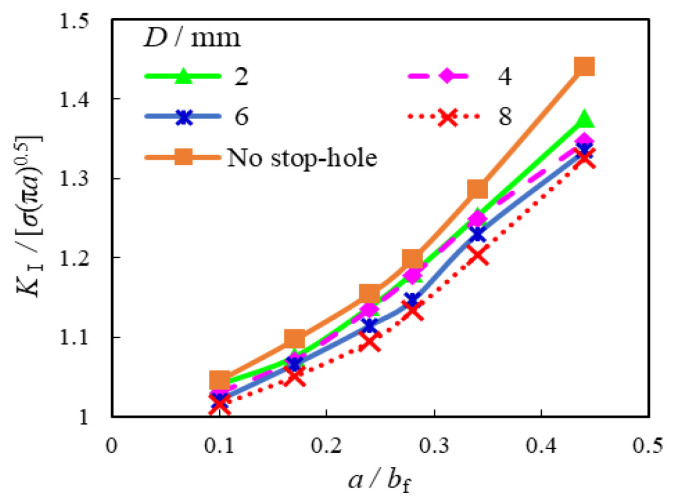
Comparison of normalized *K*_I_ with varied *a*/*b*_f_.

The circumferential stress gradients around the crack stop-hole with different *D* and *σ*_bt_ were plotted using spider charts in Figure 15. The maximum stress occurs at the prolongation seam to the edge of the outer crack stop-hole with the angle in polar coordinate of crack tip field, *θ*_p_, equal to zero. For the models without bolt prestress, it can be observed from Figure 15a,b that the circumferential stress gradients with respect to *θ*_p_ are close to each other for the repaired case i with smaller crack stop-holes, i.e., *D* = 2 mm when the cracks are increased from *a*/*b*_f_ = 0.12 to 0.2. The enlarged crack stop-holes *D* = 4 mm and *D* = 8 mm increase the circumferential stress gradients when *a*/*b*_f_ is increased from 0.12 to 0.2. When comparing the repaired case ii to the repaired case i, as shown in Figure 16a, it can be seen that the resultant circumferential stress gradients are slightly reduced for the model with *σ*_bt_ = 46 MPa as shown in Figure 15c and pronounced decreased for the model with *σ*_bt_ = 124 MPa as shown in Figure 15d. The contour area is greatly reduced for the latter case and even extended to the area, with *θ*_p_ ranging from 120° to 150° adjacent to the seam connecting two crack tips, especially for the model with *D* = 2 mm and *σ*_bt_ = 124 MPa. As a sequence, the open angle of the crack tip around the crack stop-hole can be reduced to some extent, owing to additional prestress effect from the gasket. Moreover, the effect of *D* on the circumferential stress gradients around the crack stop-hole was reduced to some extent, as evidenced from the plotted spider curves which became closer to each other in Figure 15d.

#### 3.3.3. Comparison of Stress Intensity Factor

Based on the accurate and reliable modelling of normalized SIF, as introduced previously in Section 3.2, sixty numerical models of two repair cases with crack stop-holes were analysed in this study. This was also done by adjusting a fixed variable listed below when all other variables remained unchanged and the changes of such fixed variables could be quantified properly.

Given that the geometric correction factor, *Y*(*a*), is determined by the crack size in Equation (1), the influence of the *a*/*b*_f_ on the numerical *K*_I_ normalized by *σ*_bf_√(π·*a*) was analysed first. Figure 16 shows the relationship between the normalized *K*_I_ and *a*/*b*_f_ with 0 ≤ *D* ≤ 8 for the repair case i. The slopes of all curves of normalized *K*_I_ are generally conformable to a nonlinear increase when *a* is in a certain range. However, with the increase of *a*/*b*_f_, all the plotted resultant curves with crack stop-holes increased continuously below these of the original unrepaired model with constantly lower slope, especially when *a*/*b*_f_ is greater than 0.3. Therefore, decreased stress intensity factor *K*_1_ and a slower crack growth rate can be expected for the corrugated plate girders repaired by crack stop-holes.

**Figure 17:**
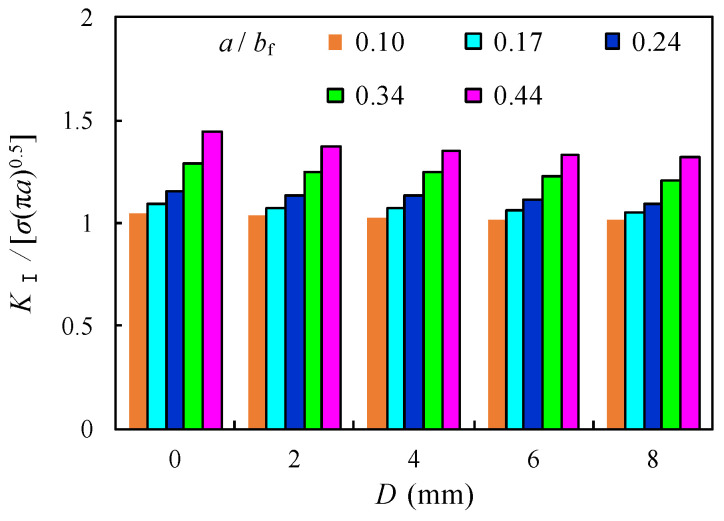
Comparison of normalized *K*_I_ with varied *D*.

The relationship between the normalized *K*_I_ and *D* with the crack propagation ratio 0.1 ≤ *a*/*b*_f_ ≤ 0.44 for the repair case i is shown in Figure 17 and Table 5. Although the normalized *K*_I_ were observed to increase with the increase of the crack propagation in the flange, they slightly decreased with the increase of *D* and became comparatively greater as the crack ran deeper. For instance, the reduction of the normalized *K*_I_ was nearly 2.2% and 3.8% for the model with *a*/*b*_f_ = 0.17 and 0.44, respectively, when *D* increased from 2 mm to 8 mm. This is similar to aforementioned comparison of *K*_t_, which means the enlargement of the size of the crack open-hole has limited influence on the further reduction of SIF and slowing down the crack propagation.

**Figure 18:**
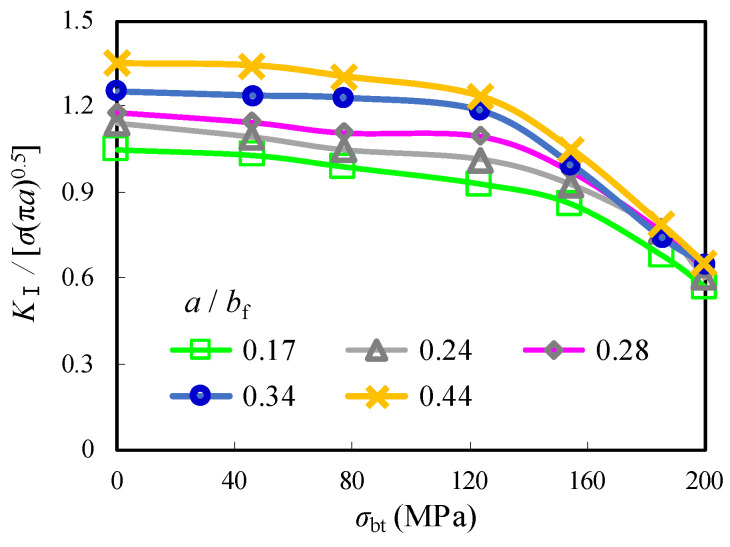
Effect of *σ*_bf_ on normalized *K*_I_ with varied *a*/*b*_f_.

The relationship between the normalized *K*_I_ and *σ*_bt_ with the crack propagation ratio 0.1 ≤ *a*/*b*_f_ ≤ 0.44 and *D* = 2 mm for the repair case ii is shown in Figure 18 and Table 5. It was observed that the normalized *K*_I_ remains stably and linearly decreased as *σ*_bt_ is increased until 124 MPa and significantly drops to nearly 0.55 as *σ*_bt_ is equal to 0.55. This can be attributed to a shift from the original tensile area around the edge of the crack open-hole that was prone to fatigue cracking to a compression-oriented area with the introduction of *σ*_bt_. As a result, the compression induced by *σ*_bt_ not only compensates for the subsequent tensile stress on the flange plate but also creates a seal between the steel plate surface and the bolt gasket prevents cracks from propagation, thus achieving the purpose of repair. Furthermore, the increment of the normalized *K*_I_ with increased *a*/*b*_f_ was decelerated when the enlarged prestress of the gasket from the high-strength bolt was applied. Taking the crack propagation from *a*/*b*_f_ = 0.17 to *a*/*b*_f_ = 0.44, for example, the normalized *K*_I_ increased by 30.3% and 32.7% for the models with *σ*_bt_ = 46 MPa and 124 MPa, respectively, while such increments were notably reduced to 22.2% and 14.1%, respectively, for the models with *σ*_bt_ = 154 MPa and 200 MPa. It can be indicated that higher prestress of the gasket from the high-strength bolt is even beneficial for the repair case ii containing longer crack in the joints of corrugated plate girders. The comparison of *K*_I_ given above is comparable to the maximum stress results of the high-strength bolt crack stop-hole, as reported in Ref. [16]. In this referred research, the hole edge stress at the tension area hole of about 1.5 mm was reduced to nearly 82.6% of that of the only stop-hole model. Meanwhile, the stress at the surface of steel plate decreased by nearly 103% as a result of the conversion of tensile stress to compressive stress. Therefore, the beneficial effect of prestress of the high-strength bolt for repair was also confirmed in this referred work. 

**Figure 19:**
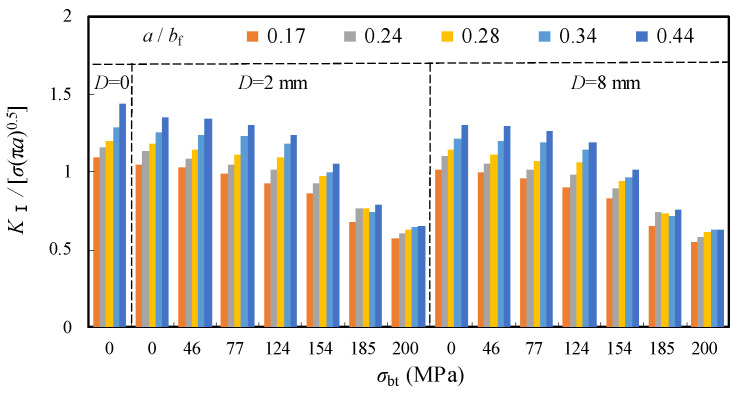
Comparison of normalized *K*_I_ with varied *σ*_bt_.

The above-mentioned relationships between the normalized *K*_I_ and *σ*_bt_ for the repair case ii were further compared with a supplement of two contrast groups of the unrepaired case (i.e., *D* = 0) and *D* = 6 mm, as shown in Figure 19. It can be observed that the normalized *K*_I_ of the original unrepaired model was greatly reduced with enlarged *D* and increased *σ*_bt_, in which the latter is obviously superior to the former. Taking the model with *a*/*b*_f_ = 0.34, for example, the normalized *K*_I_ decreased by 3.4% and 47.7% for the models with identical rate increased *D* (from 2 mm to 6 mm) and *σ*_bt_ (from 46 MPa to 200 MPa), respectively. 

## 4. Concluding Remarks

The distinct stress gradient of corrugated plate girders repaired by crack stop-holes was evaluated through numerical analysis. The contributions of geometric characteristics of the crack stop-hole and the added bolt prestress to the stress intensity factor as a metric for fracture were investigated. The conclusions can be drawn as follows.

1. The developed numerical finite element model was validated against test results in terms of strain distribution and critical stress for the cracked joints in the corrugated plate girder. It also reaches a good agreement with theoretical prediction of geometric correction factor of cracked flange plate allowing for eccentricity of crack propagation seam.
2. The presence of crack open-holes eliminated the plastic regions near the crack tip and obviously reduced the local stress concentration of the joint between the longitudinal fold and the inclined fold of the corrugated web. The corresponding stress concentration factor was greatly reduced by 34% for the model with a moderate-sized open-hole, which became less obvious for the model with an oversized open-hole.
3. With the introduction of the prestress of the crack stop-hole through bolt preloading, the reduction of the local stress concentration was nearly 50% with the prestress increased to 46 MPa, but such a reduction is inconspicuous for higher prestress. Relatively high circumferential stress gradients and the crack open angle of oversized crack stop-holes can be decreased owing to additional prestress effect from the gasket.
4. The corrugated plate girders repaired by crack stop-holes are demonstrated to have decreased stress intensity factor and slower crack growth rate than original unrepaired case. The enlargement of crack open-hole has limited influence on the reduction of stress intensity factor and crack propagation. In contrast, higher bolt prestress was more beneficial for the consistent reduction of stress intensity factor of the model with a crack open-hole, even containing long crack.

## Figures and Tables

**Table 1:** Mechanical properties of test materials [11].

Type	Chemical Composition (%)					Mechanical Properties		
	C	Si	Mn	P	S	Yield Stress (MPa)	Ultimate Stress (MPa)	Elongation (%)
Steel plate	0.16	0.33	1.36	0.035	0.022	480	582	25
Welding rod	0.15	1.15	1.85	0.025	0.025	420	502	22
Bolt/gasket	-	-	-	-	-	940	1030	9

**Table 2:** List of relative error related to *K*_I,FE_/*K*_I,Theoretical_.

*a*/*σ*_bf_	*b*_l_/*b*_bf_				
	0.16	0.49	0.66	0.79	0.85
0.12	1.01	1.01	1.01	1.01	1.02
0.24	1.01	1.01	1.01	1.01	0.98
0.34	0.99	1.00	1.00	1.02	1.05
0.40	0.99	1.00	1.00	1.01	1.05

**Table 3:** Geometric range of cracked joints in modelling.

Parameter	Symbol	Unit	Analysis Range	Interval
longitudinal fold width	*b*_l_	mm	16~85	33, 16, 13, 7
Diameter of crack stop-hole	*D*	mm	0~8	2
Bolt prestress load	*P*_b_	kN	0~0.8	0.3
Converted bolt prestress	*σ*_bt_	MPa	0~124	0, 31, 46
nominal crack length	*a*	mm	10~20	3

**Table 4:** List of *K*_t_ with varied *σ*_bt_ and *a*/*b*_f_.

*a*/*b*_f_	*K*_t_							
	D = 0	2 mm				4 mm	6 mm	8 mm
	*σ*_bt_ = 0	0	46 MPa	77 MPa	124 MPa	0		
0.10	4.59	3.13	2.25	2.10	2.12	2.16	1.68	1.42
0.17	4.19	3.28	2.17	2.14	2.14	2.36	1.83	1.53
0.24	3.76	3.25	2.16	2.13	2.13	2.41	1.87	1.56
0.28	3.55	3.23	2.20	2.10	2.08	2.40	1.82	1.50
0.34	3.25	3.00	2.17	2.06	1.94	2.29	1.76	1.44
0.44	2.94	2.76	2.18	2.08	1.86	2.16	1.62	1.32

**Table 5:** List of *K*_I_/[*σ*_bf_√(π·*a*)] with varied *σ*_bt_ and *a*/*b*_f_.

*a*/*b*_f_	*K*_I_/[*σ*_bf_√(π·*a*)]							
	*D* = 0	2 mm				4 mm	6 mm	8 mm
	*σ*_bt_ = 0	0	46 MPa	77 MPa	124 MPa	0		
0.10	1.05	1.04	1.03	1.00	0.99	1.03	1.02	1.02
0.17	1.10	1.05	1.03	0.99	0.93	1.07	1.07	1.05
0.24	1.15	1.14	1.09	1.05	1.01	1.14	1.11	1.09
0.28	1.20	1.18	1.15	1.11	1.10	1.18	1.15	1.13
0.34	1.29	1.25	1.24	1.23	1.19	1.25	1.23	1.20
0.44	1.44	1.38	1.34	1.30	1.23	1.35	1.34	1.33

## Data Availability

All data generated or used during the study are available from the corresponding author by request.

## Abbreviations

*a*: *a*	nominal crack length and a half crack length respectively
*a*_0_, *a*_1_, *a*_2_	constants for Westergaard function
*b*_1_, *b*_f_	longitudinal fold width and flange width respectively
*C*	counter clockwise contour initiating at lower crack surface and terminating on upper crack surface
*D*	diameter of crack stop-hole or controlled radius of SIF dominance zone around the crack tip
*E*	Young’s modulus
*e*	eccentricity between the centre of the crack and the centreline of the flange
*F*	applied test load at midspan
*J*	amount of energy released per unit area of crack surface increase
*K*_I_	stress intensity factor for crack plane normal to direction of tensile loading
*K*_t_	stress concentration factor
*P*_b_	bolt prestress load
*r*	mode I crack with the circle of radius
SIF	stress intensity factor
T	traction vector
*υ*	Poisson’s ratio
*σ*, *ε*	stress tensor and strain tensor respectively
*σ*_bf_	longitudinal tensile stress on bottom flange
*σ*_bt_	converted bolt prestress
*σ*_max_	Maximum principal stress around crack stop-hole
*σ*_nom_	nominal stress in flange plate with crack stop-hole
*ζ*	value that makes Z singular
*u*	displacement vector
*θ*_c_	corrugation angle
*θ*_p_	angle in polar coordinate of crack tip field
*W*	elastic strain energy density
*x*_ij_	transverse distance from flange centreline
*Y*(*a*)	dimensionless geometric correction factor
*Z*	Westergaard function
*z*	a base Cartesian coordinate for the Hamiltonian transformation
*γ*	correction variables
